# Long lasting complete molecular remission after suspending dasatinib treatment in chronic myeloid leukemia

**DOI:** 10.1186/2162-3619-1-17

**Published:** 2012-07-11

**Authors:** Klára Gadó, András Matolcsy, Judit Csomor, Dóra Kicsi, Csaba Bödör, Gyula Domján

**Affiliations:** 1 1st Department of Internal Medicine, Semmelweis University, Korányi S. Street 2, 1083, Budapest, Hungary; 2 1st Department of Pathology and Experimental Cancer Research, Semmelweis University, Budapest, Hungary; 3 Department of Internal Medicine, St Rokus Hospital, Budapest, Hungary

**Keywords:** Chronic myeloid leukemia, Dasatinib, Imatinib, Tyrosine kinase inhibitor, Drug intolerance

## Abstract

Tyrosine kinase inhibitors specific for BCR-ABL, were a major breakthrough in CML therapy. Second generation tyrosine kinase inhibitors (dasatinib, nilotinib) are indicated for imatinib resistant and intolerant patients. Present guidelines recommend continuous drug dosing for maintaining remission. There is no available data concerning the optimal duration of dasatinib therapy. We report the case of an imatinib intolerant patient who succeeded a complete molecular remission with dasatinib. Dasatinib was stopped bacause of intolerance, but complete molecular remission was sustained for one year and minor molecular remission for 27 months after discontinuation of dasatinib.

## Summary

Tyrosine kinase inhibitors specific for BCR-ABL, were a major breakthrough in CML therapy. Second generation tyrosine kinase inhibitors (dasatinib, nilotinib) are indicated for imatinib resistant and intolerant patients. Present guidelines recommend continuous drug dosing for maintaining remission. There is no available data concerning the optimal duration of dasatinib therapy. We report the case of an imatinib intolerant patient who succeeded a complete molecular remission with dasatinib. Dasatinib was stopped bacause of intolerance, but complete molecular remission was sustained for one year and minor molecular remission for 27 months after discontinuation of dasatinib.

The introduction of tyrosine kinase inhibitors specific for BCR-ABL, were a major breakthrough in CML therapy [1]. Approximately 30% of patients receiving imatinib as first-line therapy will discontinue treatment by 5 years because of imatinib resistance or drug toxicity [2].

Second generation TKIs (dasatinib, nilotinib) are indicated for those patients who are refractory or intolerant to imatinib [3,4]. However, after stopping TKI administration, relapse comes about inevi, so the present guidelines recommend continuous drug dosing [5]. While there is an ongoing prospective study with imatinib (Stop Imatinib study) [6], no available data arising from controlled studies exists with dasatinib concerning the optimal duration of therapy. Consequently, discontinuation of dasatinib is not recommended outside of clinical trials but remains an active area of research [7].

**Figure 1:**
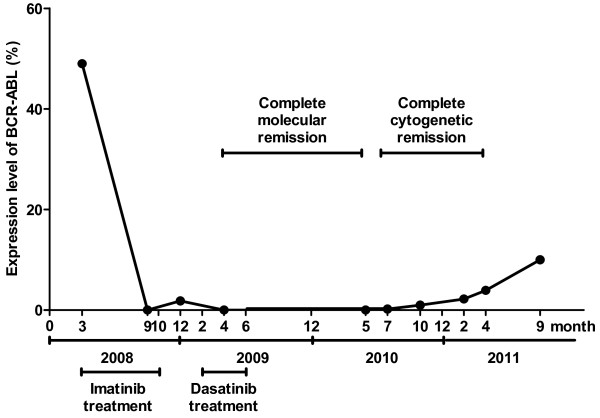
Expression level of BCR-ABL major fusion gene at various time during therapy measured by real-time quantitative PCR using ABL gene amplification as a control.

Our patient, a 84 year old lady with a chronic phase CML was intolerant to imatinib. She was given dasatinib as second line treatment. Though a reduced dose of 50 mg daily was applied, a complete molecular remission developed within two months. After four months she suspended taking the drug because of intolerance (pleural effusion, congestive heart failure, Adams-Stokes syndrome had been developed), but complete molecular remission was lasting till one year after stopping dasatinib. The patient did not receive any kind of anti-CML therapy during this year. After one year without any TKI, the expression level of *BCR/ABL* major fusion gene has increased gradually, but even now, 27 months after stopping dasatinib treatment, the patient is still in minor molecular remission (Figure1).

Because of her several kind of comorbidities, her good quality of life, the actual hematological remission and also her reluctance to take any kind of TKIs, our therapeutical strategy is only watch and wait.

This is the first report about maintaining complete molecular remission after one year of suspending dasatinib treatment.

## Abbreviations

CML, Chronic myeloid leukemia; TKI, Tyrosine kinase inhibitor.

## Competing interests

The authors have no relevant conflict of interests.

## Authors' contributions

KG, DK and GD were responsible for the clinical guidance of the patient, Judit Csomor, András Matolcsy and Csaba Bödör carried out the molecular biological examinations. All authors participated in drafting and critically revising the manuscript. All authors read and approved the final manuscript.
